# The Perception of Residency Experiences Among Orthopaedic Surgery Residents in the United States Differs by Race and Gender

**DOI:** 10.7759/cureus.81670

**Published:** 2025-04-03

**Authors:** Chrystina L James, Ryan Sanii, Johnny Kasto, Kai Zhu, Gabriel Burdick, Bushra Fathima, Tahsin Rahman, Stephanie Muh

**Affiliations:** 1 Department of Orthopaedic Surgery, Henry Ford Health System, Detroit, USA; 2 Department of Orthopaedic Surgery, University of Southern California, Los Angeles, USA; 3 Department of Neurosurgery, Yale School of Medicine, New Haven, USA

**Keywords:** diversity, orthopaedic surgery, residency experience, residency perception, survey

## Abstract

Introduction: Women and racial minorities remain underrepresented in orthopaedic surgery. While there is extensive research into the recruitment of these groups into the field, as well as more recent research regarding their representation in academic medicine and research, there is limited data on their experiences during residency. The purpose of this study is to assess the perceptions of orthopaedic surgery residents regarding their experiences during residency.

Methods: In mid-2022, a voluntary survey was sent to 2,122 orthopaedic surgery residents addressing mentorship, access to opportunities, and “fit” within their residency programs. Responses were compared by race and gender, with 345 responses received, yielding a response rate of 16.3%.

Results: Compared to male and Caucasian residents, female and underrepresented in medicine (URM) residents reported feeling less satisfied with the training they received, felt less supported, and perceived greater difficulty for women and minorities in being promoted within orthopaedics. Female residents also reported having less mentorship, receiving less recognition for their accomplishments, and being less satisfied with their career choice than male residents.

Conclusions: The results of this study highlight the need to improve equity and inclusion within orthopaedic surgery residencies in order to continue advancing diversity in the field.

## Introduction

Orthopaedic surgery is frequently identified as the least diverse specialty in medicine. Females have made up a majority of medical students since 2019 and represented 55.7% of the matriculants to MD-granting medical schools in the 2022-2023 academic year [1,2]. Racial diversity among medical students has also increased in recent years, with white students making up less than half of all medical students since 2020 [2]. Despite these trends, diversity in orthopaedic surgery has been slow to change. In 2022, females made up 7-8% of orthopaedic surgeons and 18.3% of orthopaedic surgery residents. Additionally, 72.8% of orthopaedic residents identified as White, 13.9% as Asian, and only 5.7% as Black or African American [2-4]. Recent studies have shown that racial diversity among orthopaedic surgery residents significantly decreased between 2006 and 2015, while the number of residency programs without a single underrepresented minority (URM) resident significantly increased from 2002 to 2016 [5,6].

Numerous factors play a role in the specialty’s slow progress toward gender and racial parity [7-11]. Both female and URM orthopaedic surgeons report experiencing frequent microaggressions in the workplace [11-14]. Female surgical trainees in male-dominated fields also experience a high level of gender bias and discrimination, with many reporting they may leave medicine or retire early because of gender bias, and that they would not recommend their field to others [8]. Many females are also concerned about pregnancy, maternity leave, and lactation policies, particularly during residency, as well as the well-documented increased risk of complications in pregnant surgeons [15-17]. The “hidden curriculum,” defined as the “often unrealized transmission of implicit beliefs, attitudes, and behaviors,” is likely also a contributing factor, along with those already discussed, to the persistent belief that the culture of orthopaedic surgery is not accepting of females and those who are underrepresented in medicine [9].

Mentorship is essential in recruiting and retaining diversity in orthopaedic surgery. Seeing faculty role models of similar demographics may help show students and residents that they, too, can fit into the field and be successful [18]. Studies have shown that URM medical students at institutions with higher URM representation among orthopaedic surgery residents and faculty were significantly more likely to apply to orthopaedic surgery residency, and that the number of URM faculty is significantly associated with the number of URM residents [19,20]. Additionally, it has been demonstrated that females value gender diversity within a specialty more than males, and that orthopaedic surgery residency programs with more female faculty members tend to have a higher percentage of female residents [19,21,22]. Programs that provide mentorship and early exposure to students who may not otherwise get it, such as the Perry Initiative and Nth Dimension, have been successful in helping students from diverse backgrounds apply to and match into orthopaedic surgery [23,24].

There is an abundance of research examining the recruitment of females and minorities into orthopaedic surgery residency, but there is limited data on their experience once they are actually there. Studies show that female and URM medical students have concerns about the culture of orthopaedics, but it has not been shown if this perception persists among those who become orthopaedic surgery residents [10,18,25]. The primary aim of this study was to assess how orthopaedic surgery residents view their residency experience, including program culture/“fit,” mentorship, research, and patient care opportunities, and to determine if these views differ by race or gender.

## Materials and methods

A voluntary survey was sent to all orthopaedic surgery residents listed in the American Orthopaedic Association (AOA) Council of Orthopaedic Residency Directors (CORD) contact list. The survey aimed to assess orthopaedic surgery residents’ experiences with their residency regarding its culture, mentorship, research, and patient care opportunities. It was initially distributed to 2,122 residents in March 2022, and a final reminder email was sent two months later to those who had not yet completed the survey to maximize the response rate. The survey was anonymous, and participants were informed of the voluntary nature of their participation, with an emphasis on confidentiality.

Survey content

Demographic information, including post-graduate year, race, ethnicity, sex, and residency program location, was self-reported. The survey used a five-point Likert scale to assess 17 questions regarding equity in residency training, including relationships with faculty, mentorship, career goals, financial resources, research opportunities, participation in surgical cases, participation in patient care, training satisfaction, program culture, promotion opportunities based on gender and race, faculty member representation based on gender and race, recognition of accomplishments, and specialty choice satisfaction. Inclusion in the analysis was limited to those who completed at least one survey item beyond the demographic questions. Participants who completed only demographic data and did not answer any of the survey questions were excluded.

Responses were assigned numerical values as follows: strongly disagree = 1, disagree = 2, neutral = 3, agree = 4, strongly agree = 5. We then calculated the average response to each question, stratified by gender (male or female) and race (Asian, White, URM). For the purposes of this study, we considered race responses of Black or African American, Native Hawaiian or Pacific Islander, and Other to be URM, consistent with the American Association of Medical Colleges (AAMC) definition of URM [26].

Statistical analyses

All statistical analyses were performed using IBM SPSS Statistics for Windows, Version 26 (Released 2019; IBM Corp., Armonk, New York). Patient responses were grouped into cohorts stratified by sex and race. Student’s t-tests were utilized to compare male versus female average responses to each survey item. Analyses of variance (ANOVA) were used to compare average scores for each survey item between racial groups. P-values were reported for all statistical analyses, with significance defined as p < 0.05.

This study was reviewed and approved by the Institutional Review Board (IRB) of Henry Ford Health. An IRB number was not obtained, as this was an IRB-exempt study.

## Results

This survey was sent to 2,122 orthopaedic surgery residents in the United States. A total of 390 survey responses were recorded for an initial response rate of 18.4%. Of these, 45 respondents completed only the demographics section and were excluded, yielding a total sample size of 345 and a final response rate of 16.3%.

Gender distribution was 67.2% male (n = 232) and 32.8% female (n = 113). Racial and ethnic distribution was 76.5% White (n = 264), 11.9% Asian (n = 41), and 11.6% URM (n = 40). Of those considered URM, 17 identified as Black or African American, 2 as Native Hawaiian or other Pacific Islander, and 21 as Other. Residency program location was geographically concentrated in the Northeast (35.7%, n = 123) and Midwest (29.0%, n = 100), with smaller representation in the Southeast (16.8%, n = 58), West (10.4%, n = 36), and Southwest (8.1%, n = 28). Post-graduate standing of respondents was 14.5% PGY-1 (n = 50), 13.0% PGY-2 (n = 45), 16.8% PGY-3 (n = 58), 25.5% PGY-4 (n = 88), and 29.6% PGY-5 (n = 102); 0.6% (n = 2) did not respond to this question.

Responses to each question by gender are shown in Table 1. Female residents had a lower level of agreement than male residents in response to the statements: “I have a faculty member who helps me achieve my career goals” (4.04 ± 0.98 vs. 4.27 ± 0.87, p = 0.015); “I feel supported by my program to achieve my career goals” (4.19 ± 1.01 vs. 4.37 ± 0.80, p = 0.036); and “My participation in surgical cases is equal or better compared to other residents in my program who are my year” (3.54 ± 1.08 vs. 3.79 ± 1.01, p = 0.018). Female residents also agreed less than males that the culture of their residency program allows people of all backgrounds to contribute to decision-making (4.05 ± 1.08 vs. 4.42 ± 0.77, p < 0.001), that they fit in with the culture of their program (4.04 ± 1.13 vs. 4.36 ± 0.82, p = 0.004), that they feel recognized for their accomplishments in residency (3.52 ± 1.20 vs. 3.76 ± 0.94, p = 0.032), and that they are satisfied with their specialty choice (4.42 ± 0.87 vs. 4.69 ± 0.56, p = 0.001).

**Table 1:** Resident Perceptions by Gender Data presented as mean±SD. P-values less than 0.05 are considered significant and bolded.

Question	Average Response ± 95% Confidence Interval		t-Test Values
	Male	Female	p-value
I have a faculty mentor who helps me achieve my research goals	4.09 ± 0.95	3.96 ± 1.01	0.121
I have a faculty mentor who helps me achieve my career goals	4.27 ± 0.87	4.04 ± 0.98	0.015
I feel supported by my program to achieve my career goals	4.37 ± 0.80	4.19 ± 1.01	0.036
I have access to financial resources that help me achieve my research goals	3.84 ± 1.03	3.75 ± 1.03	0.23
I have equal or better research opportunities compared to other residents in my program	3.45 ± 1.17	3.33 ± 1.13	0.182
My participation in surgical cases is equal to or better compared to other residents in my program who are in my year	3.79 ± 1.01	3.54 ± 1.08	0.018
I am satisfied with the training I have received in my program	4.35 ± 0.71	4.07 ± 1.00	0.001
There is a shortage of gender-concordant mentorship opportunities for female residents at my institution	2.50 ± 1.27	3.54 ± 1.40	<0.001
There is a shortage of race-concordant mentorship opportunities for minority residents at my institution	2.79 ± 1.30	3.72 ± 1.32	<0.001
The culture of my program allows people of all backgrounds to contribute to decision-making	4.42 ± 0.77	4.05 ± 1.08	<0.001
My residency program is a good cultural fit for me	4.36 ± 0.82	4.04 ± 1.13	0.004
I feel it is more difficult for women than men to be promoted in orthopaedics	2.82 ± 1.21	3.99 ± 1.05	<0.001
I feel it is more difficult for racial minorities to be promoted in orthopaedics	2.80 ± 1.28	3.94 ± 1.05	<0.001
There is adequate representation of racial minorities as faculty members at my institution	3.10 ± 1.24	2.43 ± 1.32	<0.001
There is adequate representation of female minorities as faculty members at my institution	3.32 ± 1.23	2.61 ± 1.45	<0.001
I feel recognized for my accomplishments in residency	3.76 ± 0.94	3.52 ± 1.20	0.032
I am satisfied with my specialty choice	4.69 ± 0.56	4.42 ± 0.87	0.001

Regarding race and gender specifically, female residents had a higher level of agreement than males that their institution has a shortage of gender- and race-concordant mentorship opportunities (3.54 ± 1.40 vs. 2.50 ± 1.27, p < 0.001 and 3.72 ± 1.32 vs. 2.79 ± 1.30, p < 0.001, respectively), and a lower level of agreement that their institution has adequate representation of female and minority faculty members (2.61 ± 1.45 vs. 3.32 ± 1.23, p < 0.001 and 2.43 ± 1.32 vs. 3.10 ± 1.24, p < 0.001, respectively). Females also had a higher level of agreement that it is more difficult for women and racial minorities to be promoted within orthopaedics (3.99 ± 1.05 vs. 2.82 ± 1.21, p < 0.001 and 3.94 ± 1.05 vs. 2.80 ± 1.28, p < 0.001).

Responses to each question by resident race are shown in Table 2. In assessing responses by race, URM and Asian residents had lower levels of agreement than Caucasian residents with the statements “I am satisfied with the training I have received at my program” (4.05 ± 1.15 vs. 4.05 ± 1.00 vs. 4.33 ± 0.72, p = 0.029) and “My residency program is a good cultural fit for me” (3.95 ± 1.38 vs. 4.10 ± 1.14 vs. 4.32 ± 0.82, p = 0.036). Asian residents had the highest level of agreement, followed by URM and then Caucasian residents, that it is more difficult for women (3.73 ± 1.05 vs. 3.30 ± 1.38 vs. 3.11 ± 1.28, p = 0.013) and racial minorities (3.80 ± 1.23 vs. 3.50 ± 1.30 vs. 3.02 ± 1.31, p < 0.001) to be promoted within orthopaedics. URM residents had lower levels of agreement than Asian and Caucasian residents, trending toward statistical significance, with the statements “I feel supported by my program to reach my career goals” (4.03 ± 1.21 vs. 4.22 ± 0.91 vs. 4.36 ± 0.80, p = 0.058) and “The culture of my program allows people of all backgrounds to contribute to decision-making” (4.05 ± 1.32 vs. 4.12 ± 0.95 vs. 4.36 ± 0.80, p = 0.052).

**Table 2:** Resident Perceptions by Race URM: underrepresented minority. Data presented as mean±SD. P-values less than 0.05 are considered significant and bolded.

Question	Average Response ± 95% Confidence Interval			f-Value Values
	URM	Asian	Caucasian	p-value
I have a faculty mentor who helps me achieve my research goals	3.78 ± 1.23	4.12 ± 1.01	4.07 ± 0.92	0.169
I have a faculty mentor who helps me achieve my career goals	3.98 ± 1.25	4.17 ± 0.83	4.23 ± 0.86	0.241
I feel supported by my program to achieve my career goals	4.03 ± 1.21	4.22 ± 0.91	4.36 ± 0.80	0.058
I have access to financial resources that help me achieve my research goals	3.78 ± 1.19	3.98 ± 0.91	3.79 ± 1.03	0.552
I have equal or better research opportunities compared to other residents in my program	3.50 ± 1.26	3.63 ± 1.16	3.36 ± 1.14	0.322
My participation in surgical cases is equal to or better compared to other residents in my program who are in my year	3.83 ± 1.28	3.56 ± 1.10	3.71 ± 0.99	0.513
I am satisfied with the training I have received in my program	4.05 ± 1.15	4.05 ± 1.00	4.33 ± 0.72	0.029
There is a shortage of gender-concordant mentorship opportunities for female residents at my institution	3.08 ± 1.39	2.85 ± 1.44	2.80 ± 1.39	0.508
There is a shortage of race-concordant mentorship opportunities for minority residents at my institution	3.28 ± 1.49	3.00 ± 1.47	3.08 ± 1.35	0.636
The culture of my program allows people of all backgrounds to contribute to decision-making	4.05 ± 1.32	4.12 ± 0.95	4.36 ± 0.80	0.052
My residency program is a good cultural fit for me	3.95 ± 1.38	4.10 ± 1.14	4.32 ± 0.82	0.036
I feel it is more difficult for women than men to be promoted in orthopaedics	3.30 ± 1.38	3.73 ± 1.05	3.11 ± 1.28	0.013
I feel it is more difficult for racial minorities to be promoted in orthopaedics	3.50 ± 1.30	3.80 ± 1.23	3.02 ± 1.31	<0.001
There is adequate representation of racial minorities as faculty members at my institution	2.88 ± 1.44	3.22 ± 1.37	2.83 ± 1.27	0.21
There is adequate representation of female minorities as faculty members at my institution	2.98 ± 1.39	3.05 ± 1.47	3.11 ± 1.32	0.825
I feel recognized for my accomplishments in residency	3.60 ± 1.32	3.56 ± 1.21	3.72 ± 0.96	0.582
I am satisfied with my specialty choice	4.58 ± 0.81	4.44 ± 0.87	4.63 ± 0.64	0.241

## Discussion

It is well-established that diversity in orthopaedic surgery is lagging behind other medical and surgical fields, from the residency level all the way through the highest levels of national societies [4,6,27,28]. Females and minorities remain vastly underrepresented throughout all levels of the field. While there has been much research examining proposed reasons for these disparities on a specialty-wide level, there is much less investigation into the day-to-day experiences of female and URM residents in their residency programs. To the authors’ knowledge, this is the first study assessing racial and gender differences in residents’ experiences and feelings of “fit” within their program.

This study demonstrates that, compared to male residents, female residents did not feel as strongly that they had a faculty mentor to help them achieve their career goals. They felt more strongly than males, however, that there was inadequate female and minority faculty representation at their institutions, as well as a shortage of race- and gender-concordant mentorship opportunities.

The importance of mentorship in medicine is well-documented, particularly among those who are underrepresented. Female, URM, and Asian residents agreed more strongly that it is more difficult for women and racial minorities to be promoted in orthopaedics. The number of female and URM orthopaedic surgeons in leadership positions is disproportionately low and may present yet another barrier to residents of the same demographic groups who do not have role models that look like them in positions of power [27,29].

Just as important as representation in orthopaedic surgery, but much harder to quantify, is “fit.” Having similar interests and experiences allows for more conversation and bonding over these similarities or for opportunities to spend time together outside of work. Being the same gender as one’s attending allows for a debrief and continued discussion in the locker room after a case. While these may seem like small things, these personal and professional conversations can lead to deeper connections, mentorship, research opportunities, and more, and they are not equally accessible to all residents. This study showed that female and minority residents felt less strongly that their residency program was a good cultural fit for them and were less satisfied with the training they had received compared to male and Caucasian residents. They also did not feel as supported by their program in achieving their career goals and had a lower level of agreement that the culture of their programs allowed people of all backgrounds to contribute to decision-making. Female residents, specifically, were less likely to agree than males that they felt recognized for their accomplishments and were satisfied with their career choice.

A recent study examining rates of resident attrition across all surgical subspecialties from 2001-2018 found that orthopaedic surgery had the highest rates of unintended attrition of female and URM residents of any specialty. Specifically, female residents had a relative risk of unintended attrition of 2.09 compared to male residents, and Black/African American residents represented 20% of residents experiencing unintended attrition despite making up only 4.1% of all orthopaedic surgery residents [30]. These alarming statistics are multifactorial but are likely contributed to by a lack of “fit,” persistent microaggressions, pervasive stereotypes and harassment, and a lack of mentorship and community.

This study is not without limitations. The largest limitation is the relatively low response rate, leading to a small sample size. Surveys were distributed via the AOA CORD email directory; however, it should be noted that this contact list is not comprehensive and does not include all orthopaedic surgery residents in the country. While the racial makeup of this study population is similar to that of orthopaedic surgery residents nationally, females are overrepresented in the study population, comprising 32.8% of respondents relative to 18.3% of all residents. This may indicate response bias, in that residents who did not feel supported by their program were more likely to respond to the survey. Lastly, it is, of course, difficult to summarize and quantify one’s personal experiences into a few short, generalized survey questions.

## Conclusions

The results of this study provide insight into the experiences of female and URM residents and demonstrate that these residents feel less supported and have less access to mentorship and other opportunities compared to male and Caucasian residents. This highlights specific areas for improvement in the ongoing efforts to increase diversity within orthopaedics. Recruiting diverse talent is important, but the present study shows that more must be done to improve equity and inclusion in the field, specifically within residency programs, to better support female and minority residents.
